# Remote ischemic conditioning enhances heart and brain antioxidant defense

**DOI:** 10.1590/1677-5449.190129

**Published:** 2020-05-08

**Authors:** Felipe Lobato da Silva Costa, Renan Kleber Costa Teixeira, Vitor Nagai Yamaki, André Lopes Valente, Sandro Percário, Marcus Vinicius Henriques Brito

**Affiliations:** 1 Universidade do Estado do Pará – UEPA, Laboratório de Cirurgia Experimental, Belém, PA, Brasil.; 2 Universidade Federal do Pará – UFPA, Belém, PA, Brasil.

**Keywords:** rats, ischemic conditioning, ischemia, reperfusion, ratos, condicionamento isquêmico, isquemia, reperfusão

## Abstract

**Background:**

Ischemia-reperfusion injury contributes to morbidity after revascularization procedures. Along with early reperfusion, tissue conditioning by alternating intervals of brief ischemia-reperfusion episodes is considered the best approach to limit tissue damage. Remote ischemic conditioning is conducted remotely, in tissues other than those under ischemia. Despite this, remote ischemic conditioning protection mechanisms are poorly understood, which can lead to misapplication.

**Objectives:**

To assess whether remote ischemic conditioning works in the heart and brain through enhancement of cells’ antioxidant defenses and whether the response is sustained or temporary.

**Methods:**

Twenty-one male Wistar rats were assigned to three groups (n = 7): SHAM: same procedure as the other groups, but no remote ischemic conditioning was carried out. RIC 10: heart and brain were harvested 10 minutes after the remote ischemic conditioning protocol. RIC 60: heart and brain were harvested 60 minutes after the remote ischemic conditioning protocol. The remote ischemic conditioning protocol consisted of 3 cycles of 5 min left hindlimb ischemia followed by 5 min left hindlimb perfusion, lasting 30 min in total. Heart and brain samples were used to measure the tissue antioxidant capacity.

**Results:**

Remote ischemic conditioning increased heart and brain antioxidant capacity after 10 minutes (0.746 ± 0.160/0.801 ± 0.227 mM/L) when compared to SHAM (0.523 ± 0.078/0.404 ± 0.124 mM/L). No enhancement of heart or brain antioxidant capacity was detected 60 minutes after remote ischemic conditioning (0.551 ± 0.073/0.455 ± 0.107 mM/L).

**Conclusions:**

Remote ischemic conditioning temporarily enhances heart and brain antioxidant defenses in male Wistar rats.

## Resumo

**Contexto:**

A lesão de isquemia e reperfusão contribui para a morbidade após procedimentos de revascularização. Juntamente com a reperfusão precoce, o condicionamento tecidual através de breves episódios de isquemia e reperfusão é considerado a melhor abordagem para limitar o dano tecidual. Apesar disso, os mecanismos do condicionamento isquêmico remoto são pouco compreendidos, o que pode levar a uma aplicação incorreta.

**Objetivos:**

Avaliar se o condicionamento isquêmico remoto funciona no coração e no cérebro através do aprimoramento da defesa antioxidante das células e se é uma resposta sustentada ou temporária.

**Métodos:**

Vinte e um ratos Wistar foram divididos em três grupos (n = 7): SHAM, no qual não foi realizado condicionamento isquêmico; RIC 10, no qual 10 minutos após o protocolo de condicionamento isquêmico, foi realizada a coleta dos órgãos; e RIC 60, no qual 60 minutos após o protocolo de condicionamento isquêmico, foi realizada a coleta dos órgãos. O protocolo de condicionamento isquêmico remoto consistiu em três ciclos de 5 minutos de isquemia, seguidos de 5 minutos de perfusão no membro posterior esquerdo, com duração total de 30 minutos. Amostras foram usadas para medir a capacidade antioxidante do tecido.

**Resultados:**

O condicionamento isquêmico remoto aumentou a capacidade antioxidante do coração e do cérebro após 10 minutos (0,746 ± 0,160/0,801 ± 0,227 mM/L) quando comparado ao SHAM (0,523 ± 0,078/0,404 ± 0,124 mM/L) . Sessenta minutos após o condicionamento isquêmico remoto, não foi detectado aumento da capacidade antioxidante do coração ou do cérebro (0,551 ± 0,073/0,455 ± 0,107 mM/L).

**Conclusões:**

O condicionamento isquêmico remoto melhora temporariamente as defesas antioxidantes do coração e do cérebro em ratos Wistar.

## INTRODUCTION

Ischemia-reperfusion syndrome is the main contributor to mortality and morbidity after revascularization procedures.^1^ In addition to early reperfusion, tissue conditioning by alternating intervals of brief ischemia-reperfusion episodes is currently the best approach to limit tissue damage.^2^

Tissue conditioning techniques can be applied locally, before or after a major ischemic period, through direct intermittent artery clamping. It has been demonstrated that these techniques are effective for reducing ischemia-reperfusion induced injury in several organs.^3^^-^5 However, since they require direct access to the artery to be occluded, they also involve the drawback of increased operating time.

These conditioning strategies can also be applied to tissues other than those exposed to ischemia. This concept has been called remote ischemic conditioning (RIC), where brief episodes of repetitive ischemia-reperfusion applied to a limb induce remote protection of other organs against potentially lethal ischemia-reperfusion injury.^6^^-^8

Remote ischemic conditioning can be administered before a planned ischemic insult, such as an elective interventional procedure (remote ischemic preconditioning);^9^ during an unplanned ischemic insult, such as endovascular thrombolysis (remote ischemic perconditioning);^10^ or after a planned or unplanned ischemic event (remote ischemic postconditioning).^11^ Since it is a low-cost, minimally invasive technique (it does not require direct access to the occluded artery) and can be easily administered during endovascular procedures, clinical trials were soon initiated.^12^

Despite its clinical applications, the underlying mechanisms of RIC-induced protection are barely understood.^13^ It has been proposed that the short IR cycles in remote tissues may provoke release of humoral factors that are sensed by the innervation of the remote organ and spread through the circulation, leading to a systemic response modulated through the parasympathetic nervous system, sending an effector signal to other organs.^14^^,^^15^

This effector signal would then activate specific receptors in cell membranes and trigger cellular survival mechanisms, in which signal transducer and activator of transcription proteins would lead to protection against ischemia-reperfusion injury.^16^ Mitochondrial protection appears to represent the final elements in the cellular protection pathway.^17^ Enhancement of antioxidant defenses in abdominal organs has been demonstrated as a protection mechanism; however, although tested, the pathway of the antioxidant-induced protection is unknown.^18^

The rapid translation from experimental studies to clinical application, despite the poorly understood mechanisms involved, could lead to misapplication of RIC. This scenario could contribute to incomplete use of the potential RIC-induced protection and this promising technique could even become lost in translation. In this study, we evaluated whether remote ischemic conditioning enhances heart and brain antioxidant defenses and whether the protection is temporary or sustained.

## METHODS

Twenty-one male Wistar rats (12-15 weeks old), with no veterinary diseases, weighing 270-300g, were used in this study. The animals were kept in a vivarium in the Experimental Surgery Laboratory at the Universidade do Estado do Pará (UEPA), Brazil, with controlled temperature, light, humidity and noise. Water and food were provided ad libitum. They were kept in collective polyurethane non-sterile cages containing 3 or 4 animals each, with sterile wood shavings as bedding material. No environmental enrichment of cages was provided.

The study followed the rules set out in Brazilian national legislation on animal care (Law: 11.794/08), which is based on NIH guidelines, and complied with the Council for International Organization of Medical Sciences ethical code for animal experimentation and the ARRIVE guidelines. The project was approved in advance by the Animal Use and Care Committee at the UEPA (protocol 01/13).

Animals were randomly assigned to three groups (N=7 rats in each group, based on a previous study),^18^ as follows: 1. sham group (SHAM): In this group, the same surgical procedure was performed as in the other groups, but no remote ischemic conditioning was carried out; 2. Remote ischemic conditioning group – 10 minutes (RIC 10): In this group, remote ischemic conditioning was performed and no organ ischemia was induced. Heart and brain were harvested 10 minutes after the end of the RIC protocol; 3. Remote ischemic conditioning group – 60 minutes (RIC 60): In this group, remote ischemic conditioning was performed and no organ ischemia was induced. Heart and brain were harvested 60 minutes after the end of the RIC protocol.

All surgical procedures were performed in anesthesia (ketamine hydrochloride and xylazine hydrochloride 70 mg/kg and 10 mg/kg, respectively, i.p.). The remote ischemic conditioning protocol consisted of 3 cycles of 5 min left hindlimb ischemia followed by 5 min left hindlimb perfusion, lasting 30 minutes in total. Hindlimb ischemia was induced using an elastic rubber band tied around the thigh of the left leg.^7^^,^^8^

Ten or 60 minutes after the end of the remote ischemic conditioning protocol, animals were euthanized by lethal anesthetic dose (xylazine hydrochloride 200 mg/kg, i.p.) and immediately subjected to median thoracotomy and craniotomy. Heart and brain were exposed and harvested for biochemical analysis.

Tissue samples were weighed, washed with 0.9% saline solution, and homogenized in 1.15% KCl solution (1:10 weight per volume) and were then transferred to an ultrasonic cell disruptor for 5 minutes to break down all lipid membranes. Samples were kept in an ice bath to prevent viability loss.

Total antioxidant capacity was determined according to its equivalence to a potent antioxidant known as Trolox, a synthetic, water-soluble analogue of vitamin E. A method proposed by Miller et al.^19^ and modified by Re et al.^20^ was employed. Briefly, this is a colorimetric technique based on a reaction between ABTS (2,2 '-azinobis-3-ethylbenzothiazoline-6-sulfonic acid, diammonium) and potassium persulfate (K2S2O8) directly yielding the free radical cation ABTS • +, a chromophore with green/blue color and maximum absorbance at the wavelengths 645, 734, and 815nm. Addition of antioxidants to this preformed radical ABTS • +, reduces it to ABTS, in proportion to the antioxidant content of the substance added, in a time and concentration-dependent manner.^21^

This can be measured spectrophotometrically by observing the change in the absorbance read at 734nm over a given time interval. Thus, the extent of discoloration is the rate of inhibition of the radical cation ABTS • + and is determined as the total antioxidant activity of the sample, which is then calculated from its relationship to the reactivity of Trolox under the same conditions, with results expressed as micromoles per liter (mM/l).^21^ The analysis was performed by a researcher blind to the study groups.

All surgical procedures were performed on the same day, in the surgery center during the afternoon shift, supported by a veterinarian. Three animals, one from each group, were processed before starting a new cycle of procedures. The total antioxidant capacity analysis was conducted on two consecutive days.

BioEstat© 5.4 software was used. All data were expressed as means ± standard deviation. Analysis of variance (ANOVA) was conducted, followed by Tukey post-hoc test correction. The unit of analysis was each single animal. All data from all animals investigated were used. Statistical significance was assumed at p < 0.05.

## RESULTS

No animals died or showed signs of diseases during follow-up. The experiment was performed once, without replacement of animals. No side effects were observed in the animals during the experiment. No significant differences were observed between groups in terms of mean body weight (SHAM: 280 ± 37 g vs. RIC 10: 284 ± 36 g vs. RIC 60: 282 ± 33 g; p = 0.76).

**Figure 1:**
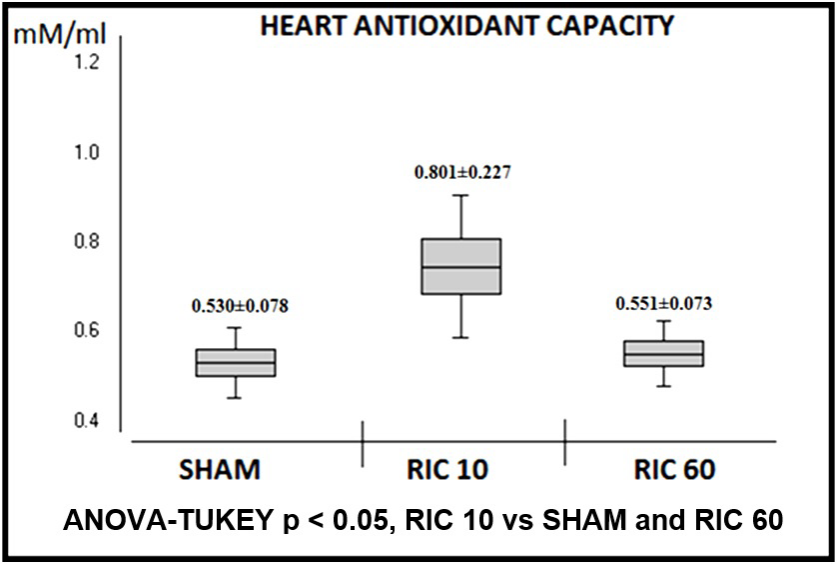
Trolox equivalent antioxidant capacity of heart, by experimental group. Mean and standard deviation.

**Figure 2:**
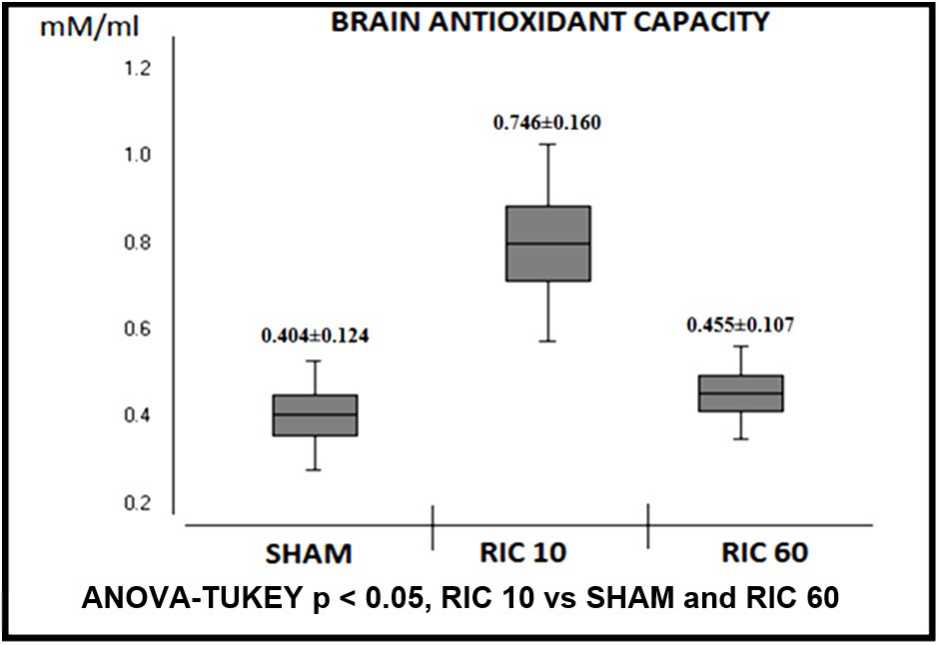
Trolox equivalent antioxidant capacity of brain, by experimental group. Mean and standard deviation.

The RIC protocol increased both heart (Figure 1) and brain (Figure 2) antioxidant capacity after 10 minutes (Heart: 0.801 ± 0.227 mM/L and Brain: 0.746 ± 0.160 mM/L) when compared to SHAM (Heart: 0.530 ± 0.078 mM/L and Brain: 0.404 ± 0.124 mM/L). In the RIC 60 group, no enhancement on heart or brain antioxidant capacity was detected 60 minutes after the RIC protocol (Heart: 0.551 ± 0.073 mM/L and Brain: 0.455 ± 0.107 mM/L).

## DISCUSSION

Remote ischemic conditioning is the most promising technique described to mitigate ischemia-reperfusion injury in many tissues, such as the myocardium, kidneys, brain, and liver, and can be easily administered during an endovascular interventional procedure.^7^^,^^8^^,^^22^^,^^23^

Induced protection mechanisms are poorly understood; however, since the technique is low-cost and minimally invasive, it quickly reached the clinical trials phase. Currently, it is being evaluated in large clinical trials, such as RECAST (Remote Ischemic Conditioning After Stroke Trial),^24^ and in other trials involving cardiovascular surgery.^23^^,^^25^

Many remote ischemic conditioning devices have been approved for clinical use. They work by delivering brief IR cycles lasting 5 minutes to the arm over a period of 40 minutes in total.^13^ These devices claim to provide protection against ischemia-reperfusion injury in situations of planned and unplanned myocardial and brain ischemia, such as patients undergoing interventional cardiothoracic procedures, and situations involving stroke or acute myocardial infarction. They are also intended to be used preventively, in chronically ill patients in the intensive care unit, to prepare for a probable ischemic event.

The rapid translation of remote ischemic conditioning to clinical practice without understanding its underlying mechanisms could lead to misapplication of this promising technique, which could even become lost in translation,^7^^-^11 i.e. the first studies in human could show no effect or negative results because the real mechanism has not been fully understood.

Indeed, our data showed that brief episodes of repetitive ischemia-reperfusion applied to a limb increased heart and brain cells’ antioxidant defenses 10 minutes after the RIC protocol, which could minimize a future IR injury. Furthermore, this is the first demonstration that remote ischemic conditioning works in the heart and brain through improvement of cells’ antioxidant defenses.

However, which exact antioxidant substances are increased was not evaluated in this study. Specific analyses for each type of antioxidant (superoxide dismutase, glutathione peroxidase, and others) should be targeted. Other measures of antioxidant activity, such as assessment of malondialdehyde, nitric oxide levels, and interleukin levels could be important in new studies to determine the pathway of RIC.

Improvement in tissue antioxidant defense was only detected 10 minutes after the RIC protocol. When we analyzed tissues 60 minutes after RIC, no improvement in total antioxidant capacity was detected. Thus, we can conclude that induced tissue antioxidant protection is not a sustained response and that remote conditioning temporarily enhances heart and brain cellular antioxidant defenses, creating a narrow window of protection.^18^ The 10 and 60-minute endpoints were chosen based on a previous study by our research team.^18^ However, more studies are necessary to determine the exact time of maximum effect and the effects of number (‘on-off’ style)^7^^,^^8^ and duration (‘dose-dependent’)^7^^,^^16^ of cycles and whether drugs or illness can modify the effect.

The mechanism underlying this enhancement in cellular antioxidant defense remains unclear. From our data, we can only state that remote ischemic conditioning induces formation of new intracellular antioxidant substances or activation of preformed antioxidant substances.^18^ Moreover, the substance involved is labile, and vanishes 60 minutes after the RIC protocol, even in the absence of reperfusion oxidative stress. The substance that promotes the enhancement of heart and brain cells’ antioxidant defenses remains unknown.

Given the narrow window of antioxidant protection enhancement, remote conditioning should be used during or very close to ischemic events or interventional procedures to achieve its maximum benefits. Preventive use for chronically ill patients, or applying the cycles long before an interventional procedure do not seem to be the best approaches for extracting the maximum benefit. Our study demonstrates that the window of antioxidant protection lasts minutes in rodent models, and there is no evidence that it would last hours in humans.

Given the short-duration of protection, it would not be beneficial to perform prophylactic cycles of remote ischemic preconditioning in a graft recipient while waiting for surgery. Rather, remote ischemic perconditioning administered to a graft recipient during the surgery seems to be the most promising technique.

The findings of our study should be interpreted within the context of its limitations. This is an animal model, and the same findings may not be applicable in their entirety to humans. Moreover, we performed RIC under anesthesia; therefore, some of the findings may have been contributed by the effects of anesthesia on the physiological state of the rats.

In summary, remote ischemic conditioning temporarily enhances heart and brain antioxidant defenses in male Wistar rats, creating a narrow window during which antioxidant protection is enhanced. RIC should be used during or very close to ischemic events or interventional procedures to achieve its maximum benefits.
